# Internalization of the Membrane Attack Complex Triggers NLRP3 Inflammasome Activation and IL-1β Secretion in Human Macrophages

**DOI:** 10.3389/fimmu.2021.720655

**Published:** 2021-09-28

**Authors:** Ines Diaz-del-Olmo, Jonathan Worboys, Fatima Martin-Sanchez, Anna Gritsenko, Ashley R. Ambrose, Gillian M. Tannahill, Eva-Maria Nichols, Gloria Lopez-Castejon, Daniel M. Davis

**Affiliations:** ^1^ Division of Infection, Immunity and Respiratory Medicine, Faculty of Biology, Medicine and Health, Lydia Becker Institute of Immunology and Inflammation, The University of Manchester, Manchester, United Kingdom; ^2^ Adaptive Immunity Research Unit, GSK, Stevenage, United Kingdom

**Keywords:** membrane attack complex, inflammasome, macrophage, IL-1β, complement, NLRP3

## Abstract

Interleukin 1β (IL-1β) plays a major role in inflammation and is secreted by immune cells, such as macrophages, upon recognition of danger signals. Its secretion is regulated by the inflammasome, the assembly of which results in caspase 1 activation leading to gasdermin D (GSDMD) pore formation and IL-1β release. During inflammation, danger signals also activate the complement cascade, resulting in the formation of the membrane attack complex (MAC). Here, we report that stimulation of LPS-primed human macrophages with sub-lytic levels of MAC results in activation of the NOD-like receptor 3 (NLRP3) inflammasome and GSDMD-mediated IL-1β release. The MAC is first internalized into endosomes and then colocalizes with inflammasome components; adapter protein apoptosis associated speck-like protein containing a CARD (ASC) and NLRP3. Pharmacological inhibitors established that MAC-triggered activation of the NLRP3 inflammasome was dependent on MAC endocytosis. Internalization of the MAC also caused dispersion of the trans-Golgi network. Thus, these data uncover a role for the MAC in activating the inflammasome and triggering IL-1β release in human macrophages.

## Introduction

Interleukin 1β (IL-1β) is a pro-inflammatory cytokine with multiple roles in inflammation. When dysregulated, however, IL-1β also underlies the pathology observed in several inflammatory diseases including toxic shock syndrome, rheumatoid arthritis, and type 2 diabetes (1). Thus, production of its active form is tightly regulated by a multi-protein complex termed the inflammasome. The best studied inflammasome is the NOD-like receptor pyrin domain-containing protein 3 (NLRP3) inflammasome and is canonically activated by the detection of two consecutive signals: a priming signal, such as exogenous lipopolysaccharide (LPS), followed by an activation signal, such as the toxin nigericin. Oligomerization of the NLRP3 inflammasome results in the recruitment of multiple proteins to the complex, including the adaptor protein ASC and the effector protease caspase 1, resulting in the activation of caspase 1 (2–4). Active caspase 1 processes gasdermin D (GSDMD) to form pores in the cell membrane and cleaves pro-IL-1β into its mature active form to be secreted from the cell (5–7).

The complement system is also activated upon recognition of damage-associated and pathogen-associated molecular patterns (DAMPs and PAMPs), such as LPS. This acts in a cascade of protein interactions which results in the formation of the membrane attack complex (MAC), also known as terminal complement complex (TCC) (8). The MAC is formed by the complement proteins C5b, C6, C7, C8 and C9 and has traditionally been studied for its ability to form pores in the membrane of pathogens and dysfunctional cells in order to clear them.

Healthy host cells have mechanisms to prevent lysis by MAC pores. During homeostasis, CD59 blocks MAC insertion into the cell membrane. However, this receptor can become exhausted during prolonged inflammation, allowing MAC pore formation (9). Additionally, nucleated cells are able to remove the MAC from the cell membrane by endocytosis, exocytosis or ectocytosis, also known as outward vesiculation (10–13). In murine cells and human epithelial and endothelial cells, sub-lytic levels of the MAC can activate caspase 1 and trigger IL-1β secretion (14–16). This is important because multiple inflammatory diseases are characterized by increased levels of both IL-1β and complement (17–20). However, it is not clear whether the MAC can directly impact inflammasome activation in human myeloid cells, a primary source of IL-1β. Here, we show that LPS-primed human macrophages internalize the MAC into EEA1+ endosomes, which leads to dispersion of the trans-Golgi network, activation of the NLRP3 inflammasome and secretion of IL-1β to the extracellular milieu.

## Materials and Methods

### Cell Culture and Differentiation

Human primary macrophages were differentiated from monocytes isolated from blood provided by the National Blood Transfusion Service (Manchester, UK) with ethical approval from the Research Governance, Ethics, and Integrity Committee at the University of Manchester (REC 05/0401/108). In brief, peripheral blood mononuclear cells (PBMCs) were isolated from leukocyte cones by ficoll gradient centrifugation. Monocytes were isolated using the human CD14 MACS separation kit (Miltenyi Biotec) and cultured at a concentration of 5 x 10^5^ cells/mL for 7 days in complete RPMI media containing RPMI-1640 (Sigma), 10% FBS (Gibco), 1% L-glutamine (Gibco), 1% penicillin/streptomycin (Gibco). Media was supplemented with 50 ng/mL M-CSF (Peprotech) for differentiation into monocyte-derived macrophages (MDMs).

The THP1 cell lines were cultured using complete RPMI media at a density of 5 x 10^5^ cells/mL and, differentiated towards a macrophage-like phenotype using 0.5 µM phorbol-12-myristate 13-acetate (PMA, Sigma) for 16 hrs. Cells were rested for 24 hrs in RPMI complete media before cell activation. THP1 wild type cells (TIB-202) were obtained from ATTC and THP1^nlrp3-/-^ cells were a gift from Prof. Veit Hornung (Gene Center Munich) (21). These cells were used to generate THP1^nlrp3-/-^/eGFP-NLRP3 cells as indicated below.

### Virus Production and Transduction of THP1^nlrp3-/-^/eGFP-NLRP3

Human NLRP3 was cloned by the Gateway cloning system in a lentiviral destination vector, pLNT-UbC-eGFP-#, generated by Dr Pawel Pazek (University of Manchester) (22). Packaging plasmids psPAX2 and pMD2.G were a gift from Didier Trono (Addgene plasmid #12260 and #12259). HEK293T cells were plated at a concentration of 3.5 × 10^5^ cell/mL for 24 hrs and transfected using Lipofectamine 2000 (Invitrogen) following the manufacturer’s instructions. In short, 8 µl Lipofectamine, 1.2 µg pMD2.G, 0.4 µg psPAX2 and 1.5 µg of pLNT-UbC-eGFP-NLRP3 were used per reaction. The following day, the media was replaced, and cells were further incubated for 2 days. Supernatants were then filtered with a 0.45-µm filter to obtain a cell-free extract of viral particles. Viral particles containing our vector of interest were used to transduce 5 × 10^4^ THP1^nlrp3-/-^ cells, with 8 µg/mL polybrene (Sigma). Cells, together with both the viral particles and polybrene, were centrifuged at 1000 g for 1 hr at 30°C. Pelleted cells were then re-suspended in fresh complete RPMI media.

### Cell Stimulation

For inflammasome activation, MDMs or THP1 cells were primed with 1 µg/mL LPS (lipopolysaccharide from Escherichia coli O26:B6, Sigma) in complete media for 3 hrs, washed with serum-free RPMI media, and treated with 10 µM nigericin for 45 mins or the Membrane Attack Complex (MAC) for the indicated time in serum free RPMI media. To form the MAC, cells were treated for 15 mins with 10 µg/mL C5b6, unless otherwise specified, and 10 µg/mL anti-CD59 mAb followed by treatment with 10 µg/mL C7, 10 µg/mL C8 and 10 µg/mL C9 (Complement Technologies). NLRP3 inflammasome activation was impaired using 1 µM MCC950 (Pepreotech). GSDMD processing was blocked using 10 µM NSA (Calbiochem). Endocytosis was blocked with 0.1 µg/mL nystatin (Merck), 10 µM cytochalasin D (Merck), or 10 µM dynasore (Calbiochem), unless otherwise specified. All inhibitors were used for 30 mins before and during stimulation with nigericin or the MAC in serum free RPMI media.

### Cell Death Assay

Cell death was established by measuring lactate dehydrogenase (LDH) release in supernatants. Upon treatment, supernatants were collected, centrifuged at 500 g to remove cell debris and LDH release was assessed using a quantitative colorimetric assay (CytoTox96^®^ Non-Radioactive Cytotoxicity Assay, Promega), following manufacturer’s instructions. Results were expressed as cell death percentage, relative to a lysis control representing 100%.

### Caspase-Glo^®^ 1 Inflammasome Assay

The activity of caspase 1 was assessed using a quantitative luminescence assay (Caspase-Glo^®^ 1 Inflammasome kit, Promega). In brief, cell supernatants were combined with the aminoluciferin substrate Z-WEHD for 1 hr and luminescence was measured. Results were expressed as fold-increase relative to vehicle-only treated cells.

### Enzyme‐Linked Immunosorbent Assay (ELISA)

Cytokine release was measured in supernatants using the Human IL-1β or IL-18 DuoSet ELISA kit (R&D) following manufacturer’s instructions.

The amount of MAC, also known as terminal complement complex (TCC), was measured in cell lysates using a human TCC ELISA kit (HycultBiotech) following manufacturer’s instructions.

### Immunoblot

Cell lysates were prepared in RIPA buffer (50 mM Tris-HCl pH 7.4, 1% NP-40, 0.25% Na-deoxycholate, 150 mM NaCl and 1 mM EDTA in miliQ dH_2_O) supplemented with protease inhibitor cocktail (Calbiochem) for 30 mins on ice. Lysates were centrifuged at 18,000 g for 20 mins at 4°C to eliminate the insoluble fraction. Protein concentration in cell lysates was determined using a Bicinchoninic Acid Protein Assay (Pierce™ BCA Protein Assay Kit, Life Technologies) and samples were diluted to an equal protein amount of 30 µg. Cell supernatants were centrifuged at 500 g for 5 mins to remove cell debris and concentrated using centrifugal cellulose filters (10 kDa MW Amicon centrifugal filter devices, Merck Millipore), as indicated by the manufacturer. Cell lysates and supernatants were diluted to 1x reducing Laemmli buffer containing 10 mM 1,4-Dithiothreitol (Sigma), heated at 95°C for 10 mins, and separated by 4-12% Bis-Tris NuPAGE gels (Invitrogen) in NuPAGE MES buffer (Invitrogen) at 165 V for 35 mins. A color protein standard (P7719, New England Biolabs or Precision Plus, Bio-Rad), was used for MW references. Proteins were transferred onto 0.2 µm PVDF membranes (GE Healthcare), blocked with 5% Bovine Serum Albumin (BSA, Sigma) in TBST (10 mM Tris-HCl, 15 mM NaCl, 0.05% Tween^®^ 20 at pH 7.5) for 1 hr at room temperature and incubated with the indicated primary antibody in blocking buffer overnight at 4°C. Membranes were washed and incubated for 1 hr at room temperature with the appropriate HRP-conjugated secondary antibody. Membranes were washed and developed using the Clarity Western ECL Substrate (Bio-Rad) and protein bands were visualized using a ChemiDoc™ MP Imager (Bio‐Rad).

The primary antibodies used for immunoblotting and their final concentrations were goat Ab anti-human IL‐1β (0.1 µg/mL, R&D Systems), rabbit Ab anti-human GSDMD (0.14 µg/mL, Novus Biologicals), rabbit mAb anti‐human caspase‐1 (1:1000, D7F10, Cell Signalling Technology), mouse mAb anti‐human NLRP3 (1 μg/mL, Cryo-2, Adipogen), mouse mAb anti‐β‐actin‐HRP (0.2 μg/mL, AC-15, Sigma). HRP conjugated secondary antibodies used were rabbit Ab anti‐goat‐HRP (0.13 µg/mL, Sigma), goat Ab anti‐rabbit‐HRP (1:3000, Bio-Rad), and goat Ab anti‐mouse‐HRP (1:3000, Bio-Rad).

### Confocal Imaging

Cells were cultured in chambered coverglasses (Nunc™ Lab-Tek™ II, Thermo Scientific™) at a concentration of 2 x 10^5^ cells/mL. Cells were differentiated, primed with LPS and activated with the MAC or nigericin, as above, fixed with 4% PFA for 15 mins, blocked and permeabilized with 2% BSA and 0.1% Triton (Sigma) in PBS for 30 mins and stained overnight at 4°C with the indicated primary antibody in 2% BSA in PBS. Cells were washed, and matched secondary antibodies were added for 1 hr in 2% BSA in PBS when needed. Samples were then washed 3 times with PBS before being imaged. For all imaging experiments C9 was conjugated in-house with Janelia Fluor 549 NHS ester (Tocris, 6147) or Alexa Fluor 647 NHS ester (Thermo Fisher Scientific, A20006). In brief, 50 µl of C9 (1 mg/mL) was mixed with 5 µl of Janelia Fluor 549 or Alexa Fluor 647 NHS ester (1 mg/mL) in 100 µM NaHCO_3_ in PBS and incubated for 1 hr at room temperature on a rotator. The excess dye was removed using size-exclusion chromatography (7K MWCO Zeba™ Spin Desalting Column Thermo Scientific) by centrifugation at 1500 g for 2 mins. Protein concentration and degree of labelling were measured by absorption and calculated according to the manufacturer’s instructions. Imaging was performed with an inverted confocal microscope (Leica TCS SP8) using a 100x/1.40NA oil-immersion objective or a 63x/1.20NA oil-immersion objective. Excitation was performed with a pulsed white-light laser and emission was detected using time-gated HyD detectors functioning in standard mode. Images were exported and analyzed using ImageJ (23). The proportion of NIK fluorescence within the cytoplasm was calculated by subtracting the nuclear fluorescence from the cell total fluorescence and expressing this relative to the fluorescence of NIK within the entire cell. Nuclei and cell outlines were identified manually using brightfield images.

The primary antibodies used for immunostaining and their final concentrations were as follows: mouse IgG1 mAb anti-human ASC (2 µg/mL, O93E9, BioLegend), mouse IgG2a mAb anti-human TCC (5 µg/mL, aE11, Abcam), sheep Ab anti-human TGN46 (1.25 µg/mL, Bio-Rad), rabbit mAb anti-human EEA1 (1:200, C45B10, Cell Signaling Technology), rabbit Ab anti-NIK (1:200, Cell Signaling Technology). The secondary antibodies used were goat Ab anti-mouse IgG2a Alexa Fluor 488 (2 µg/mL, Invitrogen), goat Ab anti-mouse IgG1 Alexa Fluor 568 or 647 (2 µg/mL, Invitrogen), donkey Ab anti-sheep Alexa Fluor 488 (2 µg/mL, Abcam) and goat Ab anti-rabbit Alexa Fluor 488 (2 µg/mL, Invitrogen).

### Statistical Analysis

The normality of the results was analyzed by the Shapiro-Wilk normality test. Normally distributed results were analyzed by parametrical one-way ANOVA and non-normally distributed results using Friedman test. Results were expressed as the mean ± standard deviation (SD). Significant differences between samples were established where p<0.05 (*), p<0.01(**), p<0.001 (***), and p<0.0001 (****). Graphpad Prism v9 was used to analyze and graphically represent all results.

## Results

### The Membrane Attack Complex Activates the Inflammasome in Human Macrophages

It is well-established that complement deposition is highly increased in IL-1β-driven diseases and thus, we set out to test whether IL-1β secretion may be triggered from human macrophages by the terminal complement complex, the MAC. Initially, we used the human monocyte-like cell line, THP1, which was differentiated with PMA to obtain a macrophage-like phenotype. Cells were then primed with LPS for 3 hrs and treated with the complement components C5b6, C7, C8 and C9 added sequentially to form the MAC, and incubated for a further 3 hrs. To permit MAC deposition on the cell membrane, C5b6 treatment was carried out in the presence of an anti-CD59 mAb (15). As a positive control, cells were primed with LPS and then treated with nigericin (Nig) for 45 mins, a widely used potent activator for the NLRP3 inflammasome.

**Figure 1:**
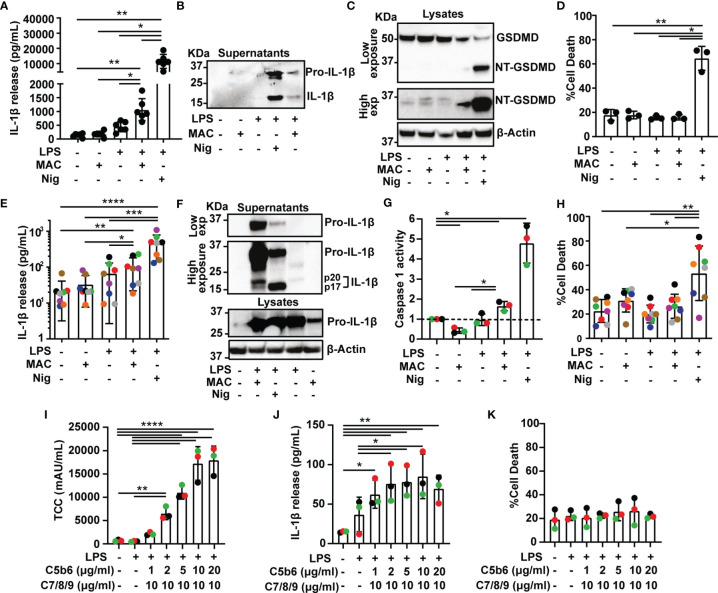
The Membrane Attack Complex induces inflammasome activation in human macrophages. **(A–K)** THP1 cells **(A–D)** and human MDMs **(E–K)** were treated with vehicles or primed with 1 µg/mL LPS for 3 hrs followed by stimulation with 10 µM nigericin (Nig) for 45 mins or with 10 µg/mL anti-CD59 mAb, 10 µg/mL C5b6, unless otherwise specified, C7, C8 and C9 (MAC) for 3 hrs. **(A, E, J)** IL-1β secretion was measured by ELISA. **(B, F)** IL-1β cleavage was analyzed in supernatants by immunoblot. **(C)** GSDMD processing was analyzed in lysates by immunoblot. **(D, H, K)** LDH release was measured as a proxy for cell death. **(F)** Pro-IL-1β production was analyzed in supernatants and cell lysates by immunoblot. **(G)** Caspase 1 activity was measured in cell supernatants and expressed as fold increase *vs* untreated cells. **(I)** TCC (MAC) amount in cell lysates was measured by ELISA. **(A, D, E, G–K)** Data is plotted as mean ± SD and is representative of three **(D, G, I–K)**, six **(A)**, seven **(E)** or eight **(H)** independent experiments. **(E, G–K)** Each color represents a matched donor. **(A, D)** Each dot represents a repeat. **(A, D, E, G–K)** Statistical significance was measured by one-way ANOVA **(A, D, G–K)** or Friedman test **(E)** (*p < 0.05, **p < 0.01, ***p < 0.001, ****p < 0.0001). **(B**, **C**, **F)** Blots are representative of two independent experiments with similar results.

Treatment with the MAC resulted in extracellular release of IL-1β (**Figure 1A**). In agreement with this, a p17 fragment corresponding with mature IL-1β was detected in cell supernatants, by immunoblotting (**Figure 1B**). Stimulation of LPS-primed cells with the MAC also led to GSDMD processing as shown by the detection in cell lysates of a p31 fragment, corresponding with the pore-forming N-terminal subunit (NT-GSDMD; **Figure 1C**). Cell death, as measured by LDH release from cells, was not significantly increased in MAC-stimulated cells, establishing that cells were not lysed by the complex (**Figure 1D**). Thus, the inflammasome can be activated in PMA-differentiated THP1 cells by the MAC.

We next set to test whether MAC-mediated inflammasome activation occurs in primary human cells. To test this, human monocyte-derived macrophages (MDMs) were activated with LPS and the MAC, which led to processing and secretion of IL-1β (**Figures 1E, F** and **Supplementary Figure 1A**). Furthermore, pro-IL-1β was also detected in lysates of MDMs treated with the MAC alone, indicating that the MAC can regulate IL-1β production (**Figure 1F**). A p20 fragment of IL-1β was also released in LPS-primed MDMs upon MAC activation which could indicate a caspase 1-independent cleavage of this cytokine (**Figure 1F**) (24). To directly test whether stimulation with the MAC triggered caspase 1 activation, treated cells were assayed for caspase 1 activity using a luminescence assay in cell supernatants. Caspase 1 activity was significantly increased upon stimulation with the MAC, as expected (**Figure 1G** and **Supplementary Figure 1B**). Similar to THP1 cells, the MAC did not increase cell death in human MDMs indicating that this complex had a sub-lytic effect (**Figure 1H**). Thus, stimulation of human MDMs with sub-lytic levels of the MAC triggered IL-1β secretion and caspase 1 activation indicating inflammasome activation.

To test the dose-dependency of MAC-mediated inflammasome activation, LPS-primed MDMs were stimulated with concentrations from 1 to 20 µg/mL of C5b6, which is the limiting component for MAC formation, in the presence of 10 µg/mL anti-CD59 and followed by 10 µg/mL of C7, C8 and C9 for 3 hrs. The amount of terminal complement complex (TCC or MAC) was measured in cell lysates by ELISA. As expected, the amount of MAC detected in cell lysates increased with increasing concentration of C5b6 (**Figure 1I**). The greatest levels of IL-1β release were observed when 10 µg/mL C5b6 was used (**Figure 1J**). Cell death was not significantly increased with any of the concentrations used, indicating that MAC formation was always sub-lytic (**Figure 1K**). Subsequent experiments were carried out with 10 µg/mL C5b6.

### MAC-Mediated IL-1β Secretion Is Dependent on Both NLRP3 and GSDMD

**Figure 2:**
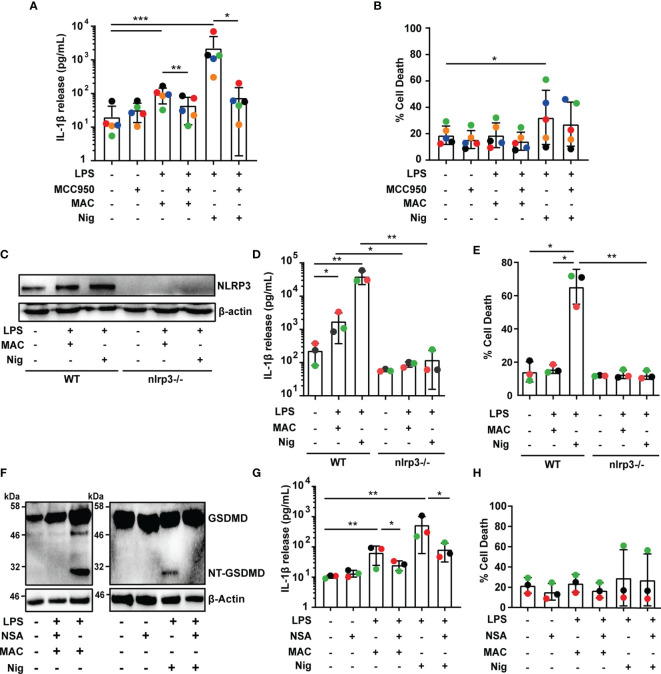
MAC-mediated IL-1β secretion is dependent on NLRP3 and GSDMD. **(A–H)** Human MDMs **(A, B, F–H)** and wild-type THP1 or THP1^nlrp3-/-^ **(C–E)** cells were treated with vehicles or primed with 1 µg/mL LPS for 3 hrs followed by stimulation with 10 µM nigericin (Nig) for 45 mins or with 10 µg/mL anti-CD59 mAb, C5b6, C7, C8 and C9 (MAC) for 3 hrs. **(A, B, F–H)** MAC treatment was carried out in the presence or absence of 1 µM MCC950 **(A, B)** or 10 µM NSA **(F–H)**. **(A, D, G)** IL-1β secretion was measured by ELISA. **(B, E, H)** LDH release was measured as a proxy for cell death. **(C, F)** NLRP3 **(C)** and GSDMD **(F)** production were analyzed in cell lysates by immunoblot. **(A, B, D, E, G, H)** Data is plotted as mean ± SD and is representative of five **(A, B)** or three **(D, E, G, H)** independent experiments. **(A, B, G, H)** Each color represents a matched single donor. **(D, E)** Each color represents a repeat. **(A, B, D, E, G, H)** Statistical significance was measured by Friedman test **(A)** or one-way ANOVA **(B, D, E, G, H)** (*p < 0.05, **p < 0.01, ***p < 0.001). **(C, F)** Blots are representative of two independent experiments with similar results.

We next set out to establish whether inflammasome activation triggered by the MAC was dependent on NLRP3. To test this, MDMs were primed with LPS and stimulated with nigericin or the MAC as mentioned previously, in the presence or absence of the NLRP3 inflammasome inhibitor MCC950 (25). The use of MCC950 significantly reduced IL-1β secretion (**Figure 2A**). In contrast, there was no statistically significant change in LDH release produced by the MAC in the presence of MCC950, establishing that cell death was not affected by the inhibitor (**Figure 2B**). For an alternative approach to test the role of NLRP3 in MAC-mediated inflammasome activation, we used THP1^nlrp3-/-^ cells, generated using CRISPR-Cas9 technology as previously described (21). The lack of NLRP3 in these cells was confirmed by immunoblotting (**Figure 2C**). Following LPS and MAC stimulation, IL-1β secretion was impaired in THP1^nlrp3-/-^ cells (**Figure 2D**). As in wild-type THP1 cells, the MAC did not trigger cell death in THP1^nlrp3-/-^ cells, evidenced by no change in LDH release (**Figure 2E**). Thus, NLRP3 is essential for IL-1β secretion triggered by the MAC in human macrophages.

Given the importance of GSDMD for IL-1β secretion upon canonical activation of the NLRP3 inflammasome and the fact that GSDMD cleavage was detected in MAC-stimulated THP1 cells (**Figure 1C**), we next investigated whether GSDMD is involved in MAC-mediated IL-1β release in MDMs. To test this, LPS-primed human MDMs were treated with the MAC in the presence or absence of the GSDMD pore inhibitor necrosulfonamide (NSA) (26). Stimulation with the MAC resulted in the cleavage of GSDMD, and GSDMD processing was impaired by NSA, evidenced by a lack of NT-GSDMD detection by immunoblotting (**Figure 2F**). Crucially, NSA-treated MDMs released significantly less IL-1β upon stimulation with the MAC (**Figure 2G**). Cell death, on the other hand, was not significantly affected (**Figure 2H**). These data are consistent with the idea that GSDMD processing, and therefore pore formation, is important for MAC-mediated IL-1β release in MDMs.

### The MAC Localizes With the NLRP3 Inflammasome in Human Macrophages

As MAC-mediated IL-1β secretion in human macrophages is dependent on NLRP3 (**Figures 2A–E**), we next set out to establish the localization of NLRP3 and the MAC within human macrophages. The MAC is formed by a single subunit of C5b6, C7 and C8 and multiple C9 subunits (27). Therefore, to visualize this complex, C9 was directly conjugated to a fluorescent dye (C9-AF647 or C9-JF549). Using confocal imaging, C9 was observed at the cell periphery, indicative of membrane localization, within minutes of stimulation with the MAC (**Supplementary Figures 2A, B**), but after 30 mins C9 was mainly detected intracellularly (**Supplementary Figures 2A, B**), indicating that the MAC was rapidly internalized upon deposition in the cell membrane.

**Figure 3:**
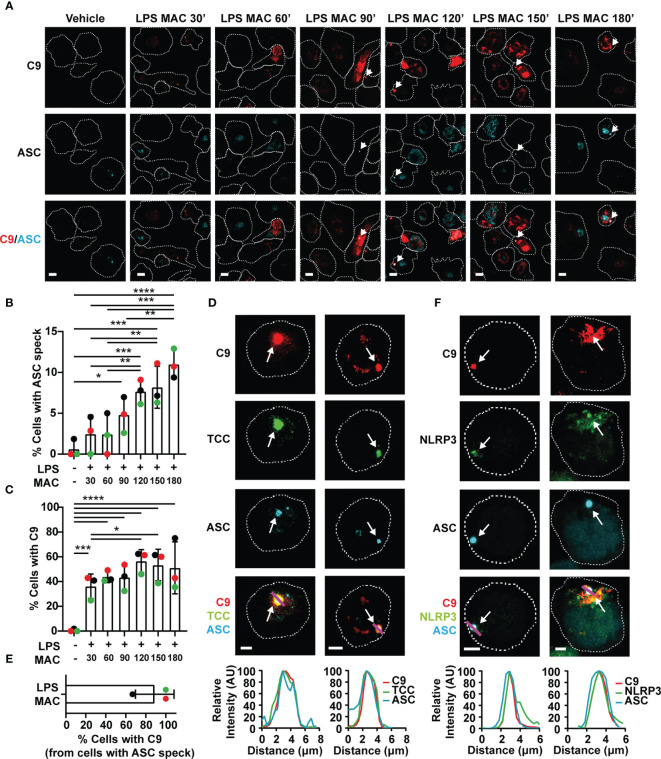
The MAC triggers inflammasome assembly and localizes to the NLRP3-ASC speck. **(A–F)** Human MDMs **(A–E)** and THP1^nlrp3-/-^/eGFP-nlrp3 cells **(F)** were treated with vehicles or primed with 1 µg/mL LPS for 3 hrs followed by stimulation with 10 µg/mL anti-CD59 mAb, C5b6, C7, C8 and C9 labelled with JF-549 (MAC) for the indicated time **(A–C)** or 3hrs **(D–F)**. **(A)** Representative confocal images of C9 (red) and ASC (cyan) overtime. **(B)** Percentage of cells with ASC specks over time. **(C)** Percentage of cells with C9 over time. **(D)** Confocal imaging of C9 (red), ASC (cyan) and TCC (green). **(E)** Percentage of ASC speck positive cells with C9 in the speck. **(F)** Confocal imaging of C9 (red), ASC (cyan) and NLRP3 (green). **(A, D, F)** Dashed lines represent the outline of the cell, arrows point to specks, magenta lines correspond to the plotted line profiles and scale bars are 10 µm **(A)** and 5 µm **(D, F)**. **(B, C, E)** Data is plotted as mean ± SD, each color represents a matched donor. **(B, C)** Statistical significance was measured by one-way ANOVA (*p < 0.05, **p < 0.01, ***p < 0.001, ****p < 0.0001). **(A–F)** Data is representative of 3 independent experiments.

Given that canonical NLRP3 activation is characterized by the oligomerization of the adaptor protein ASC into a speck, cells were stained with an anti-ASC mAb to visualize the inflammasome complex after stimulation with the MAC using C9-JF549. Confocal microscopy revealed ASC speck formation in LPS-primed MDMs after 90 mins of MAC stimulation, indicative of inflammasome assembly (**Figures 3A, B** **)**. The percentage of cells with ASC specks increased over 3 hrs following stimulation (**Figures 3A, B** **)**. Likewise, the percentage of cells with readily detectable intracellular C9 was also increased over 3 hrs (**Figures 3A, C** **)**. Surprisingly, C9 accumulated in specific regions of the cell interior and formed structures with a similar appearance to the ASC specks (**Figures 3A, D** **)**. Further research using specific markers is needed to determine the intracellular trafficking of C9, but strikingly, after 3 hrs of MAC-stimulation, ASC and C9 colocalized in the majority of cells containing ASC specks (**Figures 3D, E** **)**. The MAC is formed by multiple complement components and thus, an accumulation of C9 does not necessarily indicate that fully formed MAC structures remain present intracellularly in the macrophages. To investigate whether the fully formed MAC is internalized, MDMs were also stained with a mAb targeting the TCC. This anti-TCC mAb binds a neoepitope of the MAC, which is only present when C5b6, C7 and C8 are assembled in the complex. Indeed, this mAb also marked regions where both C9 and ASC were detected (**Figure 3D**), establishing that the complete MAC complex is internalized by macrophages. Considering that MAC formation and internalization occurs within minutes of treatment with C5b6, C7, C8 and C9 (**Supplementary Figure 2A**) and that inflammasome assembly occurs 90 mins after stimulation, this implies that the MAC triggers downstream events that over time activate the inflammasome.

To further test whether NLRP3 oligomerization could be directly triggered upon stimulation with the MAC, we generated a THP1 cell line that stably expressed eGFP-NLRP3 in a nlrp3^-/-^ background by transducing THP1^nlrp3-/-^ cells with lentiviral particles containing the vector pLNT-UbC-eGFP-NLRP3. Expression of eGFP-NLRP3 in these cells, which otherwise lacked NLRP3, restored their ability to form an active inflammasome upon stimulation with nigericin as shown by IL-18 secretion (**Supplementary Figure 3A**), increased cell death (**Supplementary Figure 3B**), and NLRP3 and ASC speck formation (**Supplementary Figures 3C, D**). Moreover, the use of the NLRP3 inhibitor MCC950 impaired IL-18 secretion, cell death and ASC speck formation (**Supplementary Figures 3A, B, D**). Stimulation with LPS and nigericin led to caspase 1 (Casp-1) and IL-1β processing (**Supplementary Figure 3E**). These results demonstrate that eGFP-NLRP3 is functionally active. More importantly, treatment of these cells with the MAC led to ASC speck formation. At the speck, there was also an accumulation of C9 and NLRP3 (**Figure 3F**). This provides further evidence that the MAC triggers ASC oligomerization and NLRP3 inflammasome assembly in human macrophages.

### The MAC Is Internalized in EEA1 Positive Endosomes

**Figure 4:**
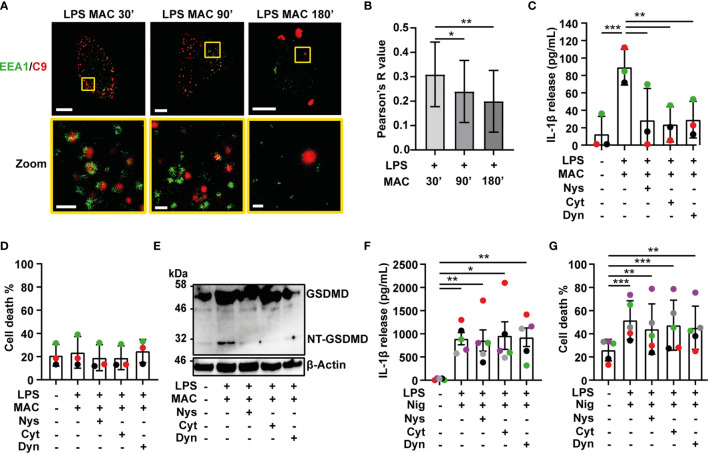
Internalization of the MAC is required for inflammasome activation. **(A, B)** LPS-primed human MDMs were treated with 10 µg/mL anti-CD59 mAb, C5b6, C7, C8 and C9 labelled with AF-647 (MAC) for the indicated time. **(A)** Confocal imaging of EEA1(green) and C9 (red). Yellow squares represent zoom regions and scale bars are 10 µm in full cell pictures and 1 µm in zoom regions. **(B)** Pearson’s correlation coefficient of C9 compared to EEA1. **(C–G)** Human MDMs were treated with vehicles or primed with 1 µg/mL LPS for 3 hrs followed by stimulation with 10 µg/mL anti-CD59 mAb, C5b6, C7, C8 and C9 for 3 hrs **(C–E)** or 10 µM nigericin (Nig) for 45 mins **(F, G)** in the presence or absence of 0.1 µg/mL nystatin (Nys), 10 µM cytochalasin D (Cyt) or 10 µM dyanasore (Dyn). **(C, F)** IL-1β secretion was measured by ELISA. **(D, G)** LDH release was measured as a proxy for cell death. **(E)** GSDMD processing was analyzed in cell lysates by immunoblot. **(C, D, F, H)** Data is plotted as mean ± SD, each color represents a matched donor and statistical significance was measured by one-way ANOVA **(B–D, G)** or Friedman’s test **(F)** (*p < 0.05, **p < 0.01, ***p < 0.001). **(A–G)** Data is representative of 3 **(A–E)** or 5 **(F, G)** independent experiments.

The observation that the MAC is internalized, led us to investigate how this complex is taken up by macrophages. To study this, LPS-primed MDMs were treated with all components of the MAC including C9-AF647. After 30, 90 or 180 mins, cells were fixed and co-stained with an Ab against EEA1 to mark early endosomes. Following 30 mins of MAC treatment, multiple C9 puncta were distributed in the cytosol of the cell (**Figure 4A** and **Supplementary Figure 2A**). These puncta were encircled by the early endosomal marker EEA1 (**Figures 4A, B** **)**. This suggests that C9 is internalized by MDMs *via* EEA1+ endosomes. At later time points, colocalization of C9 and EEA1 was reduced, concurrent with C9 accumulating in larger puncta resembling the structure of inflammasome specks (**Figures 4A, B** **)**. Thus, the MAC is initially internalized into early endosomes before being trafficked to the inflammasome.

### MAC-Mediated Inflammasome Activation Is Dependent on Endocytosis

Ion fluxes produced by pore formation in the cell membrane can trigger activation of the NLRP3 inflammasome (28). Therefore, we next investigated whether pore formation by the MAC in the cell membrane is what activates the inflammasome or alternatively, if internalization of the MAC triggers this process. To test this, LPS-primed MDMs were stimulated with the MAC for 3 hrs in the presence or absence of different endocytosis inhibitors: nystatin (Nys), cytochalasin D (Cyt) or dynasore (Dyn) (29–32). Strikingly, all three inhibitors abrogated IL-1β secretion triggered by the MAC (**Figure 4C**), while cell death was unaffected (**Figure 4D**).

Previous work showed that lysis of K562 cells treated with human serum increases in the presence of 40-100 µM dynasore (32). Here, treatment of LPS-primed MDMs with 40-160 µM dynasore, in the absence of the MAC, induced IL-1β secretion but did not trigger cell death (**Supplementary Figures 4A, B**). On the other hand, 10-20 µM dynasore did not result in cytokine secretion by itself, and did not trigger cell death, but significantly reduced MAC-mediated IL-1β release (**Supplementary Figures 4C, D**).

The NT-GSDMD fragment was not detected in cell lysates of cells treated with any of the endocytosis inhibitors (**Figure 4E**). This shows that inhibition of endocytosis reduced downstream caspase 1 activity and prevented processing of GSDMD. Endocytosis inhibitors did not impair IL-1β secretion in general, since neither IL-1β secretion nor cell death were affected by these inhibitors when cells were stimulated with nigericin (**Figures 4F, G** **)**. Thus, IL-1β secretion following complement-mediated inflammasome activation is specifically dependent on endocytosis of the MAC.

In endothelial cells, endocytosis of the MAC results in the recruitment of various proteins including NF-κB inducing kinase (NIK) that can phosphorylate and activate the NLRP3 sensor to assemble the inflammasome complex (16, 17, 33). To investigate whether NIK is involved in inflammasome activation in MDMs, cells were treated with LPS for 3 hrs, followed by the MAC incorporating C9-AF647 for the indicated time (**Supplementary Figure 5A**). Treatment with the MAC resulted in a change of localization of NIK (**Supplementary Figures 5A, B**). Specifically, the relative abundance of NIK in the cytoplasm increased from early time points after stimulation with the MAC and was decreased after 90 mins, reaching similar levels to cells treated only with LPS (**Supplementary Figures 5A, B**). Whilst NIK localization clearly changed over time, we did not detect colocalization with C9 (**Supplementary Figure 5C**) suggesting that the MAC and NIK may not directly associate.

### MAC-Internalization Results in Disruption of the TGN

**Figure 5:**
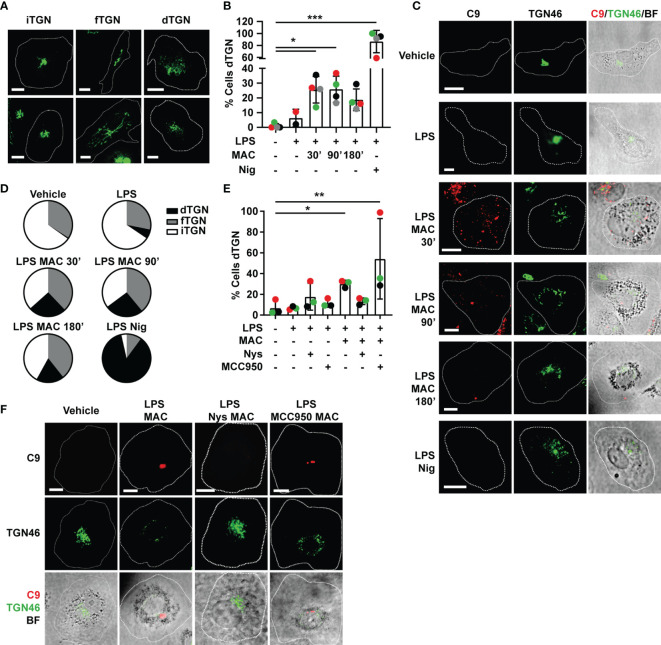
Endocytosis of the MAC triggers disruption of the TGN. **(A–D)** Human MDMs were treated with vehicles or primed with 1 µg/mL LPS for 3 hrs followed by stimulation with 10 µg/mL anti-CD59 mAb, C5b6, C7, C8 and C9 labelled with AF-647 (MAC) for the indicated time or nigericin (Nig) for 45 mins. **(E, F)** Human MDMs were treated with vehicles or primed with LPS and the MAC for 3 hrs in the presence or absence of 0.1 µg/mL nystatin (Nys) or 1 µM MCC950. **(A)** Representative confocal images of the different TGN conformations: intact TGN (iTGN), fragmented TGN (fTGN) and dispersed TGN (dTGN). **(B, E)** Percentage of cells with dTGN. **(C)** Representative confocal images of the results analyzed in panel **(B)**. **(F)** Representative confocal images of the results analyzed in panel **(E)**. **(C, F)** TGN46 (green) and C9 (red) and brightfield images (gray). Dashed lines represent cell outlines and scale bars are 10 µm. **(D)** Pie charts representing the percentage of cells with dTGN, fTGN and iTGN in each condition. **(B, E)** Data is plotted as mean ± SD, each color represents a matched donor and statistical significance was measured by Friedman’s test (*p < 0.05, **p < 0.01, ***p < 0.001). **(B–E)** Data is representative of 3 **(E, F)** or 4 **(B–D)** independent experiments.

The dispersion of the trans-Golgi network (TGN) and the recruitment of NLRP3 to the dispersed TGN have been described as upstream events to NLRP3 inflammasome assembly, with multiple inflammasome activators, including nigericin (34). Thus, we next tested if MAC-mediated inflammasome activation is also preceded by dispersion of the TGN. MDMs were treated with LPS followed by the MAC, including the labelled form of C9 (C9-AF647) as previously, for 30, 90 or 180 mins, or nigericin for 45 mins. Cells were then fixed and stained with an Ab against TGN46, a resident membrane protein of the TGN. Different structures of the TGN were revealed by this stain and thus, we classified each cell as exhibiting one of three specific conformations: (i) an intact TGN (iTGN), characterized by a compact single structure; (ii) a fragmented TGN (fTGN), characterized by various stranded structures; or (iii) a dispersed TGN (dTGN), characterized by multiple granular-like dispersed formations (examples shown in **Figure 5A**).

**Figure 6:**
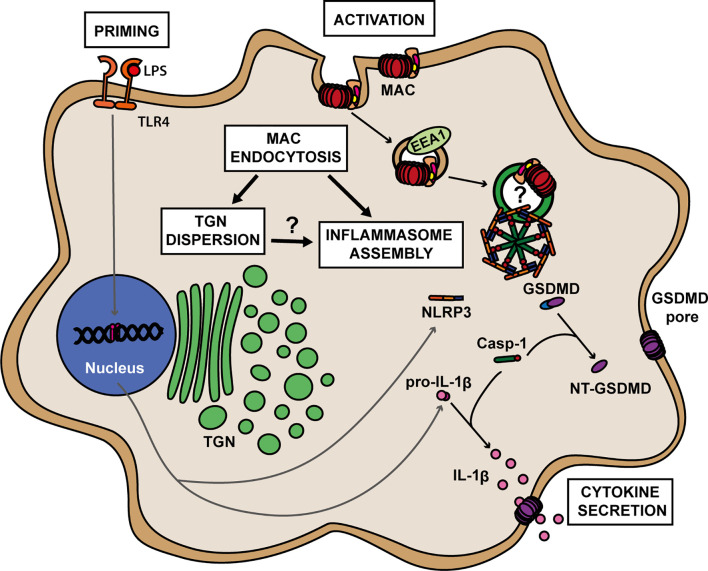
A proposed model for MAC-mediated NLRP3 inflammasome activation in human macrophages. Formation of the MAC in the cell membrane triggers its internalization *via* EEA1+ endosomes. Endocytosis of the MAC leads to dispersion of the trans-Golgi network (TGN) and to the assembly of the NLRP3 inflammasome. The roles of TGN dispersion as well as any direct involvement of MAC components on inflammasome assembly remain to be elucidated. Within the NLRP3 complex, caspase 1 (Casp-1) becomes active and cleaves GSDMD into its pore-forming subunit, NT-GSDMD, and pro-IL-1β into its active form IL-1β. Multiple NT-GSDMD subunits assemble in the cell membrane to form GSDMD pores resulting in the release of IL-1β to the extracellular milieu.

The dispersed TGN structure was observed in LPS-primed MDMs treated with either the MAC or nigericin but not in unstimulated cells (**Figures 5B, C** **)**. This establishes that either type of stimulation triggers this phenomenon for inflammasome activation, and indeed provides further evidence of the MAC to be able to activate the NLRP3 inflammasome. Except in cells stimulated with nigericin, the percentage of cells with fTGN was very similar across conditions (**Figure 5D**). The percentage of cells exhibiting a dTGN was increased in MAC-treated cells from 30 mins of stimulation, although not to the same extent as nigericin-treated cells (**Figure 5B**). The percentage of cells with dTGN remained similar after 90 mins of stimulation (25.5 ± 9% at 30 mins, 25.8 ± 18.9% at 90 mins) and was reduced by 180 mins (18.7 ± 7.3%) (**Figure 5B**). This suggests that dispersion of the TGN occurs soon after internalization of the MAC. Inhibition of MAC endocytosis using nystatin (Nys) reduced dispersion of the TGN (**Figures 5E, F** **)**. However, TGN dispersion did not decrease with the use of the NLRP3 inhibitor MCC950 (**Figures 5E, F** **)**. Thus, TGN dispersion occurs downstream of MAC internalization and upstream of NLRP3 inflammasome assembly. Together, these data establish that the MAC can be internalized by human macrophages into EEA1+ endosomes, which results in disruption of the TGN, the assembly of the NLRP3 inflammasome, caspase 1 activation, GSDMD processing and secretion of IL-1β (**Figure 6**).

## Discussion

The terminal pathway of the complement system plays an important role in the development of multiple IL-1β-mediated diseases. For instance, C5b-9 is significantly increased in the serum of patients with bacterial sepsis (19, 20) and in the plasma and synovial fluid of patients with rheumatoid arthritis and osteoarthritis (18, 35, 36). However, whether the terminal complement complex directly impacts macrophage secretion of IL-1β has not been established. Here, we show that formation of the MAC triggers NLRP3-dependent IL-1β secretion providing a link between the terminal pathway of the complement system and IL-1β release in human macrophages. As inflammation and complement activation are intricately linked, this mechanism is likely to be involved in a range of acute and chronic inflammatory diseases.

Specifically, we found that sequential addition of the complement proteins C5b6, C7, C8 and C9 in serum-free media triggered NLRP3-dependent IL-1β release in human macrophages demonstrating that the MAC can activate the inflammasome. MAC-mediated NLRP3 inflammasome activation has been previously described in lung epithelial cells, endothelial cells and myeloid cells (14–16, 37). As well as using different cell types, these prior studies used serum as a source of complement. Bioactive molecules and growth factors present in serum, such as complement component 5a or fibroblast growth factor (38, 39), could enhance MAC-mediated inflammasome responses. Thus, our data clarifies that the MAC can activate the inflammasome independently of other serum components and more importantly, that human macrophages are activated in this way, being a vital source of this cytokine in the human body.

Phagocytosis of particles opsonized by complement-containing serum can trigger IL-1β release in human macrophages (37). However, our data show that deposition of the MAC can directly trigger caspase 1 activation, GSDMD processing and IL-1β secretion in the absence of phagocytosis. This gives a broader role for this complex in promoting proinflammatory responses, including in the context of sterile inflammation when inflammasome priming is provided by endogenous DAMPs such as amyloids aggregates and cholesterol crystals (40). Moreover, the MAC triggers IL-1β secretion in the absence of cell death, suggesting that it drives macrophages into a state of hyperactivation, in which they secrete IL-1β while maintaining viability (5). Hyperactive macrophages secrete less IL-1β than macrophages undergoing pyroptosis, which fits with our observation that IL-1β secretion upon stimulation with the MAC occurs to a lesser extent than in nigericin-stimulated cells. Importantly, low levels of IL-1β are able to activate downstream signaling pathways in target cells (41–45) suggesting that the levels of MAC-mediated inflammasome activation and IL-1β release seen in our experiments can be biologically important.

Pro-IL-1β is not considered biologically active. However, proteases derived from neutrophils and pathogens such as *S. aureus* are able to process pro-IL-1β into active molecules (46, 47). Specifically, in models of acute arthritis, proteinase 3 from neutrophils can cleave pro-IL-1β (48) and MAC deposition is known to be elevated in the context of arthritis (17, 18). Thus, the high levels of pro-IL-1β secretion observed after stimulation with the MAC can be relevant both in the context of sterile inflammation and during infection.

Intracellular LPS can activate caspases 4 and 5 that in turn activate the NLRP3 inflammasome (49, 50). Here, macrophages were primed with LPS prior to MAC treatment. LPS was washed off before MAC stimulation. However, residual levels of LPS could feasibly enter the cell through the MAC and activate caspases 4 and 5. LPS from gram-negative bacteria can activate the complement cascade (51, 52). Therefore, determining whether the entrance of LPS through MAC pores leads to the activation of caspases 4 and 5 and downstream signaling pathways would be interesting to explore further in the context of infection.

In general, mechanisms by which complement activates cells are complex, multi-faceted and vary by cell type. MAC formation in human lung epithelial cells triggers increased cytosolic Ca^2+^ and loss of membrane potential in the mitochondria, with both processes being suggested to be responsible for the activation of the NLRP3 inflammasome (15). Here, we found that, in human macrophages, the MAC is internalized in EEA1+ endosomes from early time points and that endocytosis is required for inflammasome activation. This suggests that ion fluxes caused by MAC deposition in the cell surface membrane might not be sufficient to activate the inflammasome in macrophages but may trigger the internalization of the complex that in turn initiates inflammasome activation. In support of this, dispersion of the trans-Golgi network (TGN), an event that occurs with canonical NLRP3 inflammasome assembly (34), is triggered only upon internalization of the MAC. Previous work has established that other pore-forming toxins including α-haemolysin and streptolysin activate the NLRP3 inflammasome (53). These toxins are also known to be removed from the cell membrane through different mechanisms, including endocytosis (40, 54). Thus, it is possible that internalization of a pore-forming complex is, broadly, able to trigger inflammasome activation.

In response to antibody-mediated complement activation, endothelial cells internalize the MAC in Rab5+ and EEA1+ early endosomes, which results in the recruitment of NLRP3 in a process involving NF-κB–inducing kinase (NIK) (16, 17, 33). We did not observe colocalization of NIK and C9 at time points where C9 localized to early endosomes or to the inflammasome speck. However, the observation of a change in localization of NIK, going from the nucleus to the cytoplasm after stimulation with the MAC could reflect a role for NIK in MAC-mediated NLRP3 inflammasome assembly in human macrophages as shown in endothelial cells (16, 17, 33).

In retinal epithelial cells, MAC internalization in endosomes and subsequent migration to lysosomes for degradation contributes to the reduction of lytic effects of membrane-bound MAC (29). Podocytes target the MAC for degradation through the autophagic-lysosomal pathway, with the expression of the autophagy markers LC3 and p62 enhanced during this process (55). In THP1 cells, LC3, p62 and ASC are found together upon NLRP3 inflammasome stimulation (56, 57). Overall, this suggests a crosslink between the autophagic-lysosomal pathway in the NLRP3 and the MAC signaling cascades. In addition, internalization of particulate matter such as silica or alum can lead to lysosomal disruption and K^+^ efflux, leading to assembly of the inflammasome (58, 59). Given that MAC endocytosis is key to triggering inflammasome assembly in human macrophages and that the level of intracellular C9 is reduced over time, this indicates that the MAC could be targeted for degradation through the lysosomal pathway as in podocytes and retinal epithelial cells. As such, MAC internalization could result in lysosomal damage and subsequent activation of the inflammasome.

It is also possible that disruption of the TGN triggered by MAC internalization plays a role in inflammasome assembly. Endosomes constantly exchange cargo with the TGN and EEA1 can localize to the TGN in various cell types (60). In HeLa cells expressing exogeneous NLRP3 and EEA1, these proteins colocalized with the TGN protein TGN38 upon stimulation with NLRP3 activators (34) indicating the interaction between the endosomal compartment, the TGN and the NLRP3 sensor. NLRP3 can be recruited to the TGN by interaction with phosphatidylinositol-4-phosphate that is exposed in the dispersed TGN (34). In macrophages, NLRP3 activators also trigger the recruitment of mitochondria-associated endoplasmic reticulum membranes (MAMs) to the Golgi. At these MAMs, NLRP3 is phosphorylated leading to its activation (61, 62). Then, upon activation at the MAMs, NLRP3 sensors can be transported to the centrosome where inflammasome assembly occurs upon recruitment of ASC and caspase 1 (56). Considering that our findings show that dispersion of the TGN occurs before inflammasome activation, interaction of MAC-containing EEA1+ endosomes with the TGN may trigger its dispersion leading to NLRP3 recruitment and the consequent assembly of the inflammasome complex. However, further investigation using specific cell compartment markers is needed to determine precisely where the MAC locates within the cell.

These details are important because the complement system plays a role in the pathogenesis of multiple pro-inflammatory diseases characterized by increased levels of IL-1β, including septic shock and rheumatoid arthritis (17–20, 63). Direct or indirect blocking of IL-1β using drugs, such as anakinra or canakinumab, has been used in the treatment of such inflammatory diseases (1). However, the effectiveness of IL-1β blockade is context dependent, varying among patients and diseases (1). Considering that the complement system plays a role in the development of these pathologies and that the MAC triggers IL-1β secretion in human macrophages, one of the main sources of this cytokine, future therapies for IL-1β-mediated diseases could consider targeting the upstream events that trigger cytokine secretion. Specifically, the growing mechanistic evidence of how the MAC modulates inflammation through activation of the inflammasome may lay the foundations for a broader application of anti-MAC therapies in the treatment of inflammatory diseases in which the complement system and IL-1β play an important role, including osteoarthritis, rheumatoid arthritis, and diseases of the central nervous system.

## Data Availability Statement

The original contributions presented in the study are included in the article/**Supplementary Material**. Further inquiries can be directed to the corresponding author.

## Author Contributions

ID-d-O and AA: Acquisition and analysis of data. ID-d-O, JW, GT, E-MN, GL-C, and DMD: Conceptual design. ID-d-O, JW, E-MN, GL-C, DMD: Wrote the manuscript. FM-S and AG: provided novel reagents. All authors contributed to the article and approved the submitted version.

## Funding

This work was supported by a PhD studentship funded by GSK (to ID-d-O), a Wellcome Trust Investigator Award (110091/Z/15/Z), a Wellcome Trust Sir Henry Dale Fellowship (104192/Z/14/Z) to GL-C, and the Manchester Collaborative Centre for Inflammation Research (funded by a precompetitive open-innovation award from GSK, AstraZeneca, and The University of Manchester, United Kingdom). GSK was not involved in the study design, collection, analysis, interpretation of data, the writing of this article or the decision to submit it for publication.

## Conflict of Interest

Authors GT and E-MN are employed by GSK. DMD is a consultant and advisor to GSK.

The remaining authors declare that the research was conducted in the absence of any commercial or financial relationships that could be construed as a potential conflict of interest.

## Publisher’s Note

All claims expressed in this article are solely those of the authors and do not necessarily represent those of their affiliated organizations, or those of the publisher, the editors and the reviewers. Any product that may be evaluated in this article, or claim that may be made by its manufacturer, is not guaranteed or endorsed by the publisher.
