# Supplementary material for: Internalization of the Membrane Attack Complex Triggers NLRP3 Inflammasome Activation and IL-1β Secretion in Human Macrophages
Source: Front Immunol. 2021 Sep 28;12:720655. doi: 10.3389/fimmu.2021.720655 (PMC8506164; doi:10.3389/fimmu.2021.720655)
Supplement: Supplementary file 1 [file DataSheet_1.pdf]

Supplementary Figures

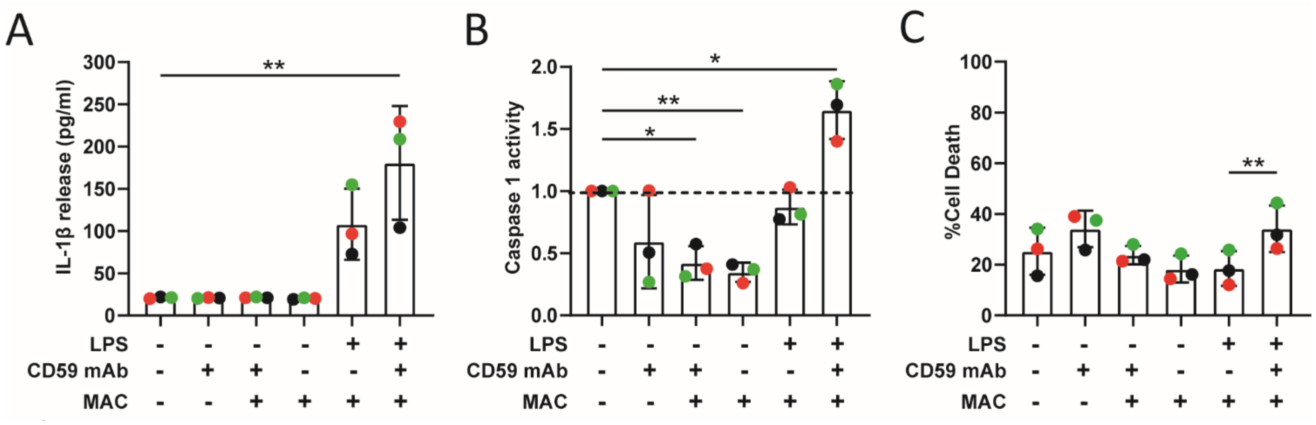

Supplementary Figure 1. LPS priming is required for MAC-mediated caspase 1 activation and mature IL-1β release. (A-C) Human MDMs were treated with vehicles or primed with 1 µg/mL LPS for 3 hrs followed by stimulation with 10 µg/mL anti-CD59 mAb or 10 µg/mL C5b6, C7, C8 and C9 (MAC) or anti-CD59 mAb and the MAC for 3 hrs. **(A)** IL-1β secretion was measured by ELISA. **(B)** Caspase 1 activity was measured in cell supernatants and expressed as fold increase vs untreated cells. **(C)** LDH release was measured as a proxy for cell death. **(A-C)** Data is plotted as mean ± SD and is representative of three independent experiments. Each color represents a matched single donor. Statistical significance was measured by one-way ANOVA (*p<0.05 **p<0.01 ***p<0.001).

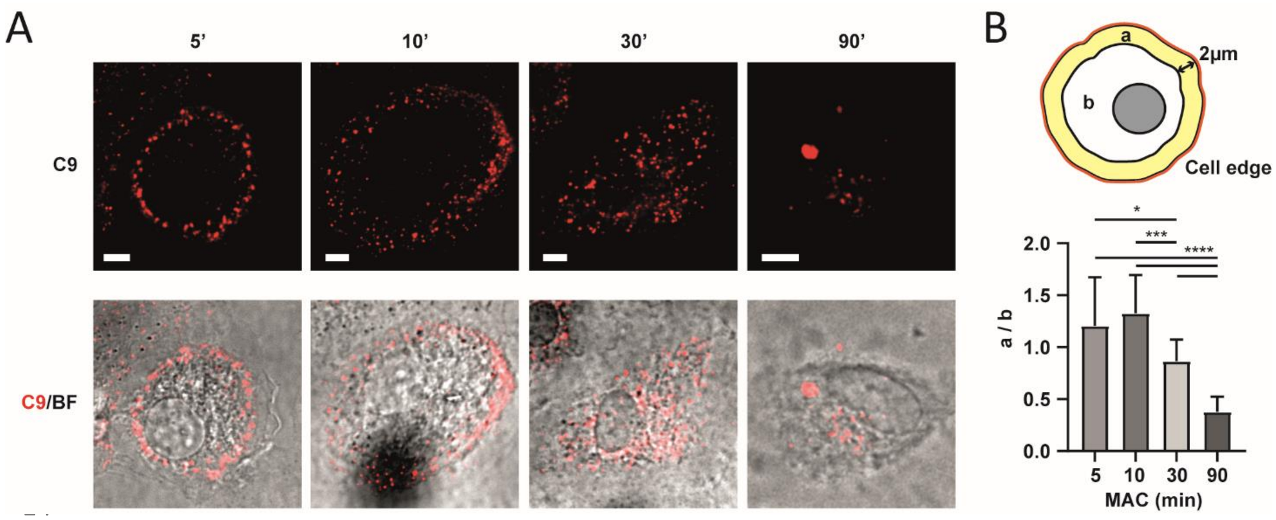

Supplementary Figure 2. The MAC is deposited in the cell membrane before internalization. Human MDMs were treated with 1 µg/mL LPS for 3 hrs followed by stimulation with 10 µg/mL anti-CD59 mAb and 10 µg/mL C5b6, C7, C8 and C9-AF647 to form the MAC for the indicated time (in minutes). **(A)** Representative confocal images of C9-AF647 (red) and brightfield (gray). Scale bars are 5 µm. (**B)** Ratio between mean fluorescent intensity of membrane C9 (a) vs intracellular C9 (b)**.** Data is plotted as mean ± SD and is representative of two independent experiments and statistical significance was measured by Kruskal-Wallis test (*p<0.05 ***p<0.001 ****p<0.0001).

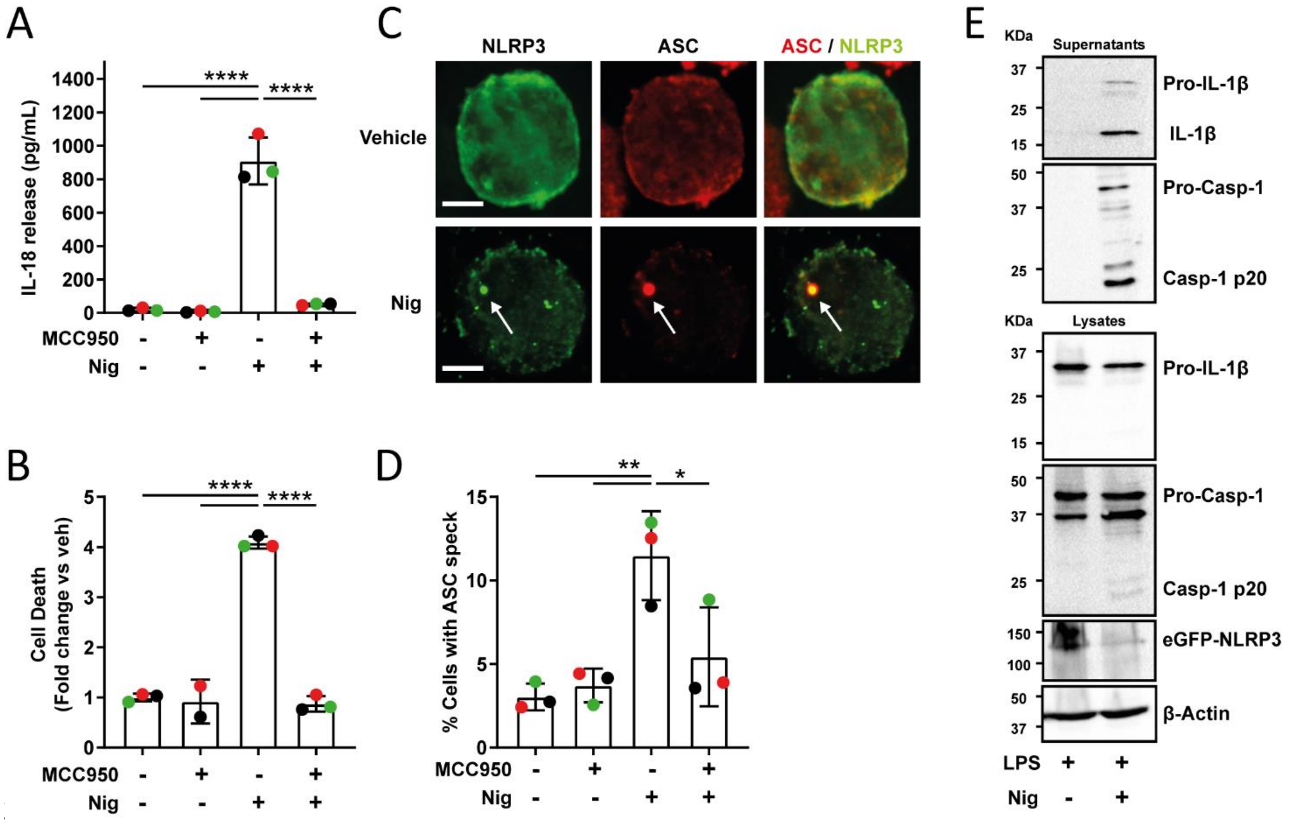

Supplementary Figure 3. $THP1^{nlrp3-/-}$/ eGFP-NLRP3 cells exhibit NLRP3 inflammasome activation. (A-E) $THP1^{nlrp3-/-}$ cells were reconstituted with eGFP-NLRP3. **(A-D)** Cells were stimulated with 10 µM Nig for 30 mins. **(A, B, D)** Nig treatment took place in the presence or absence of 10µM MCC950. **(A)** IL-18 secretion was measured by ELISA. **(B)** LDH release was measured as a proxy for cell death and represented as fold change increase vs non-treated cells (veh). **(C)** Representative confocal images of eGFP-NLRP3 (green) and ASC (red). Arrows point to specks and scale bars are 5 µm. **(D)** Percentage of cells with ASC specks. **(E)** Cells were primed with 1 µg/mL LPS for 4 hours followed by stimulation with nigericin (Nig) for 45mins. (**E)** Immunoblot of IL-1β and caspase 1 (Casp-1) in supernatants and lysates and eGFP-NLRP3 and β-actin in lysates. **(A, B, D)** Data is plotted as mean ± SD and is representative of three independent experiments. Each color represents a matched experiment. Statistical significance was measured by one-way ANOVA (*p<0.05 **p<0.01 ***p<0.001).

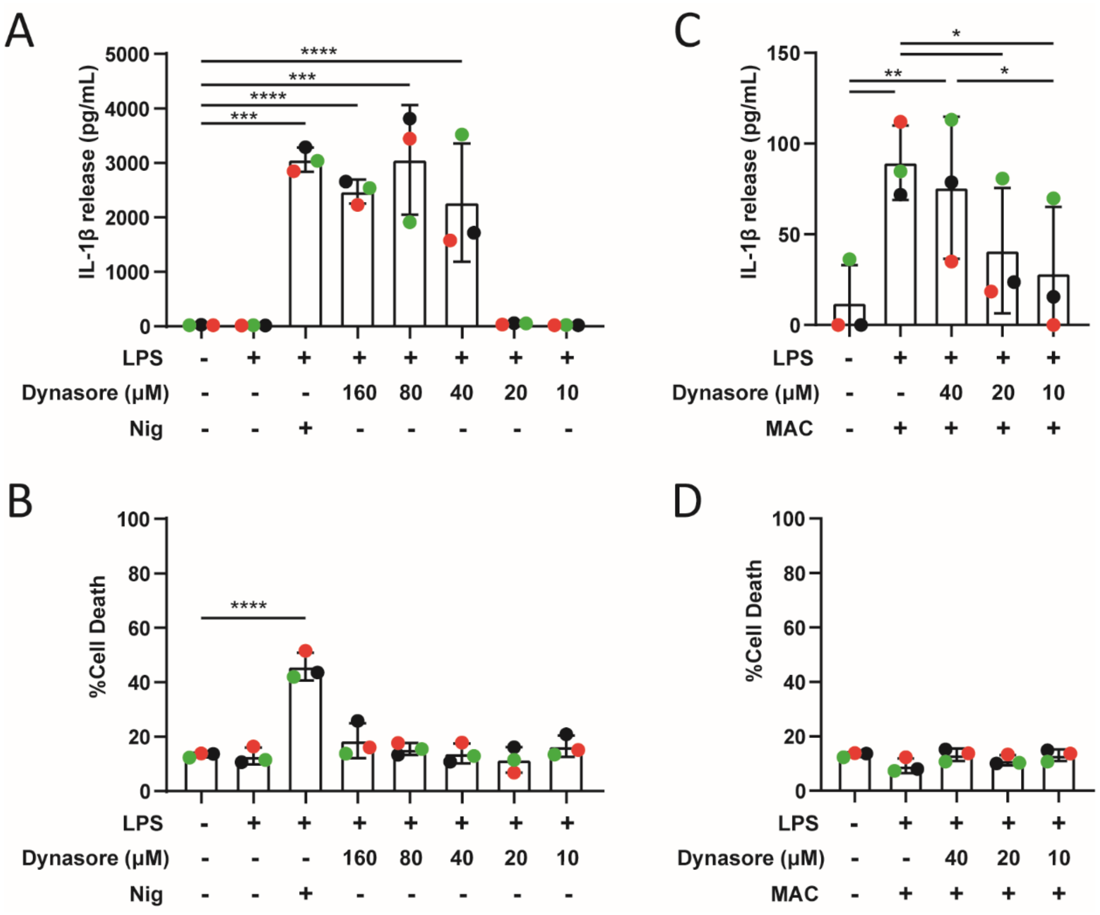

Supplementary Figure 4. Effects of dynasore in human MDMs. Human MDMs were treated with vehicles or primed with 1 μg/mL LPS for 3 hrs followed by the indicated concentration of dynasore for 30 mins. Cells were then stimulated with vehicles, Nig for 45 min (A, B) or 10 μg/mL anti-CD59 mAb or 10 μg/mL C5b6, C7, C8 and C9 (MAC) to form the MAC for 3 hrs (C, D). **(A, C)** IL-1β secretion was measured by ELISA. **(C, D)** LDH release was measured as a proxy for cell death. **(A-D)** Data is plotted as mean ± SD and is representative of three independent experiments. Each color represents a matched single donor. Statistical significance was measured by one-way ANOVA (*p<0.05 **p<0.01 ***p<0.001 ****p<0.0001).

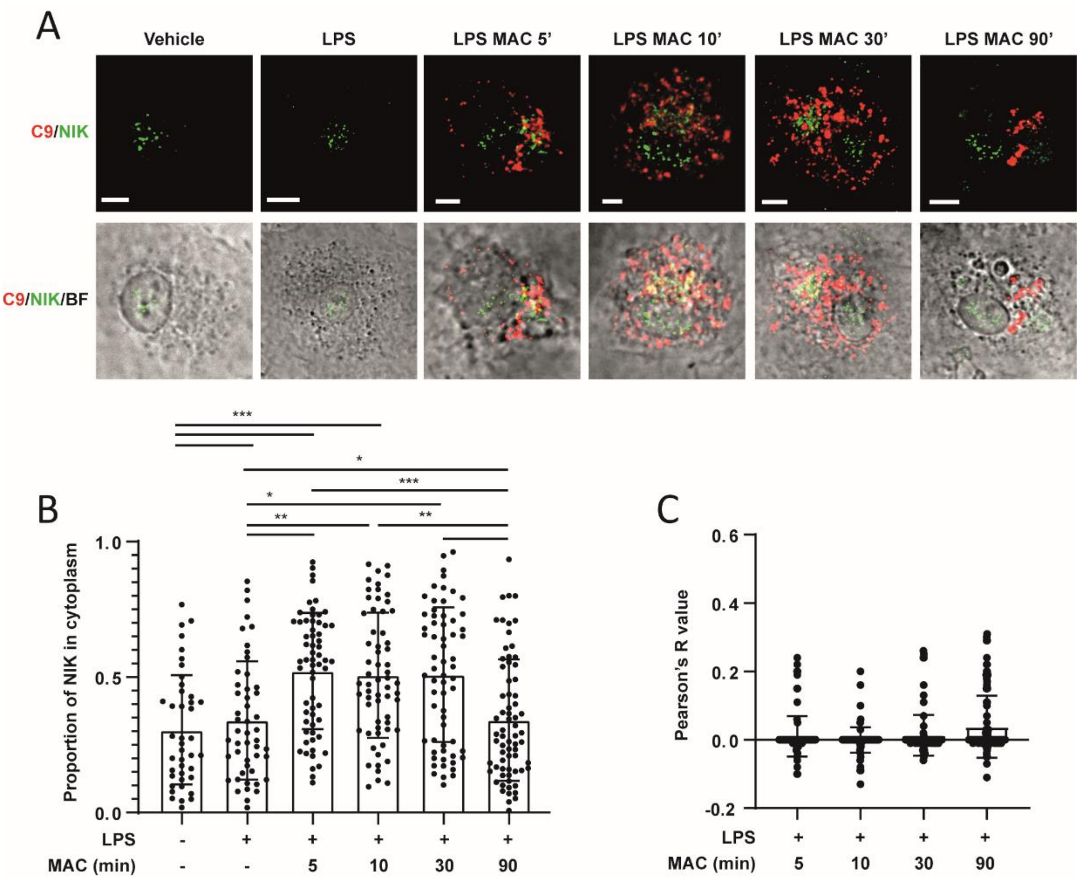

Supplementary Figure 5. NF-κB inducing kinase (NIK) localized to the cytoplasm upon stimulation with the MAC. Human MDMs were treated with 1 µg/mL LPS for 3 hrs followed by stimulation with 10 µg/mL anti-CD59 mAb and 10 µg/mL C5b6, C7, C8 and C9-AF647 to form the MAC for the indicated time. **(A)** Representative confocal images of C9-AF647 (red), NIK (green) and brightfield (gray). Scale bars are 5 µm. **(B)** Proportion of NIK within the cytoplasm overtime. **(C)** Pearson's correlation coefficient of C9 compared to NIK overtime. (**B** and **C**) Data is plotted as mean ± SD and is representative of two independent experiments and statistical significance was measured by Kruskal-Wallis test (*p<0.05 **p<0.01 ***p<0.001).
